# Evaluation of Community Restaurants Linked to Government Food and Nutrition Safety Programs: A Scope Review

**DOI:** 10.3390/foods12214009

**Published:** 2023-11-02

**Authors:** Mateus Santana Sousa, Carlos Rodrigo Nascimento de Lira, Eduardo Yoshio Nakano, Raquel Braz Assunção Botelho, Rita de Cássia Coelho de Almeida Akutsu

**Affiliations:** 1 Nutrition School, Federal University of Bahia, Salvador 40110-909, Brazil; carlos.rodrigo.n@hotmail.com (C.R.N.d.L.); rita.akutsu@gmail.com (R.d.C.C.d.A.A.); 2 Department of Statistics, University of Brasília, Brasilia 70910-900, Brazil; eynakano@gmail.com; 3 Department of Nutrition, Faculty of Health Sciences, Campus Universitario Darcy Ribeiro, University of Brasília, Brasilia 70910-900, Brazil; raquelbotelho@unb.br

**Keywords:** scoping review, evaluation, restaurants, community

## Abstract

Community restaurants linked to government food and nutritional security programs are establishments created to offer meals to the population in socially vulnerable situations. The objective was to identify the methods, approaches, criteria, and indicators used to evaluate restaurants linked to government food and nutrition security programs. A scoping review based on the Joanna Briggs Institute’s methodology and the international guide’s recommendations of preferred reporting items for systematic reviews and meta-analyses extension for scoping reviews was conducted. Medline databases via PubMed, Lilacs, Scopus, Cochrane, Web of Science, and ScienceDirect were used. Primary observational studies, systematic reviews and meta-analyses, ethnographies, documentary studies, and case studies were included, with a quantitative, qualitative, and/or mixed approach. A total of 2498 studies were identified. After taking out 180 duplicated articles, another 2202 articles were excluded by the title. Among the 71 studies selected for complete reading, 10 did not correlate with the research objective, and 12 were included after analyzing the references, totaling 73 included studies. In this review, evaluative approaches were mapped and systematized on the menu, food consumption, food health, food security and/or insecurity, nutritional education, and human right to adequate food; users’ profile and health, implantation, history, perceptions, senses, and meanings; handlers/workers; hygienic–sanitary quality; evaluation and monitoring; physical–functional planning, and rest–intake. The presented data provide elements that can be adapted in future evaluations and describe the panorama of academic production in this area.

## 1. Introduction

Community restaurants (CRs) linked to government food and nutrition security programs are world-renowned food and nutrition security establishments whose objective is the production and distribution of free or low-price meals for people in situations of social vulnerability in order to increase access to food and guarantee human rights to adequate food and fight hunger [1,2,3,4,5,6,7,8]. 

Several CR experiences can be found in countries such as Peru (Comedores Populares), Chile (Servicios de restaurantes populares), Spain (Comedores Sociales), Argentina (Comedores comunitarios), Canada (Community/collective kitchens), and Australia (Community Kitchens). Like community kitchens in Brazil, soup kitchens are located in countries such as Mexico, Peru, Uruguay, the United States of America, and Colombia [9]. 

The evaluation and monitoring of food and nutrition security are essential axes for programs and public policies to promote access to food. Through assessments, tools can be offered for improvement, management, and strengthening, especially regarding political and economic instability and threats to social rights. The evaluation must be a continuous and permanent object, aiming to reorient actions and subsidize public agents in decision making, interventions, and the implementation of public policies [10,11]. 

In this sense, there is an increase in investments by public agents in social program evaluations. However, evaluating food promotion programs is challenging due to the multiplicity of actions, multidisciplinary nature, heterogeneity of local and regional problems, and cultural and socioeconomic diversity [12]. Therefore, evaluations must be developed with knowledge and practices that may be influenced by different approaches, scientific disciplines, and theoretical–methodological traditions, which can reveal relevant evidence for the program [13,14]. 

In addition, considering that health service managers need to monitor programs to obtain information on the daily decision-making process and that population surveys are carried out but not with the desired frequency, it is necessary to develop and improve approaches, techniques, and methods for evaluation based on data produced by health information systems [15]. 

Thus, systematizing findings from CR assessments aims to improve practices and policies so that researchers can identify possible gaps and understand how such researchers have conducted studies in this area. In this way, the scoping review can help produce new evidence when the existing scientific production is recent and/or incipient and examine how research is being developed in consolidated areas, which can generate knowledge with the potential to guide decisions and actions in public policies.

The present study aims to identify the methods, approaches, criteria, and indicators used to evaluate community restaurants linked to government food and nutrition security programs.

## 2. Materials and Methods

### 2.1. Protocol and Registration

This scoping review study aims to synthesize research evidence to map the literature on a previously determined subject (nature, characteristics, and volume), identifying knowledge gaps [16]. This review had its study protocol registered in the Open Science Framework on 23 December 2022 (https://osf.io/eptfv, accessed on December 23,2022). The protocol was developed based on the recommendations of the international guide’s preferred reporting items for systematic reviews and meta-analyses extension for scoping reviews (PRISMA-ScR) [17] and the Joanna Briggs Institute (JBI) method [18].

The structure consists of six main consecutive steps: (I) identification of the question and research objective; (II) identification of relevant studies that would enable the breadth and scope of the review’s purposes; (III) study selection, according to predefined criteria; (IV) data mapping; (V) summarization of results, through a qualitative thematic analysis about the objective and question; (VI) presentation of results, identifying implications for policy, practice, or research. The acronym PCC—Population, Concept, and Context—was adopted with the following question: “What is the scientific evidence produced about the evaluation approaches carried out in community restaurants linked to governmental programs of Food and Nutrition Security?”. Therefore, they were defined based on the guiding question: population—users (target audience), professionals, and managers involved in program evaluations; concept—methods, approaches, criteria, and indicators used and evaluation results; and context—community restaurants linked to government food and nutritional security programs.

### 2.2. Inclusion Criteria

This study included observational studies (cross-sectional, case–control, cohort, and ecological studies), documentary studies, and case studies, with a quantitative, qualitative, and/or mixed approach. There was no restriction regarding publication date, language, geographic region, or country. Also included were studies that addressed the methods, criteria, and indicators used in the evaluations of CRs, carried out with users of such restaurants, professionals, and/or managers who work in these food services where eligible.

CRs linked to government food and nutritional security programs were considered to be food services financed by the municipal, state, and federal government or with public funding such as *community restaurants*, *comedores populares, comedores sociais, comedores comunitários*, *budget restaurants*, *economy restaurants*, *popular restaurant*, *community restaurants*, *government-subsidized kitchens*, *social kitchens*, *community kitchens*, *community food programs*, *soup kitchens,* and *self-service.* These are programs that offer food to the population with social vulnerability.

### 2.3. Exclusion Criteria

Studies and publications whose research context was not exclusively the evaluation of CRs, reviews, editorials, comments, perspectives, conference abstracts, reports, opinion polls, master’s theses, doctoral theses, or systematic or systematized reviews were excluded. Studies conducted in restaurants located at universities or private companies were also excluded.

### 2.4. Information Sources and Search Strategy

The last searches were conducted in June 2023 in the databases Medical Literature and Retrieval System online (Medline/via PubMed), Latin American and Caribbean Health Sciences Literature (Lilacs/via Virtual Health Library), Scopus, Cochrane, Web of Science, and ScienceDirect.

The descriptors and their synonyms were identified in the Medical Subject Headings (MeSH) and Health Sciences Descriptors (DeCS): “community restaurants”, “*comedores populares*”, “*comedores sociais*”, “*comedores comunitários*”, “budget restaurants”, “economy restaurants”, “popular restaurant”, “community restaurants”, “government-subsidized kitchens”, “social kitchens”, “community kitchens”, “community food programs”, and “soup kitchens”. These were associated with the terms referring to the evaluation, that is, “evaluate”, “*assess*”, and “*assessment*”. Along with the descriptors, the Boolean operators AND/OR were used to compose the search strategies in the databases. The strategy was specifically adapted to each database. The results were exported to the online reference manager EndNote^®^, where duplicate references were excluded. After this step, the other documents were exported to Rayyan^®^, where another evaluation of the duplicates was carried out, and the selection steps of phases I and II were carried out.

### 2.5. Data Selection and Extraction

**Figure 1:**
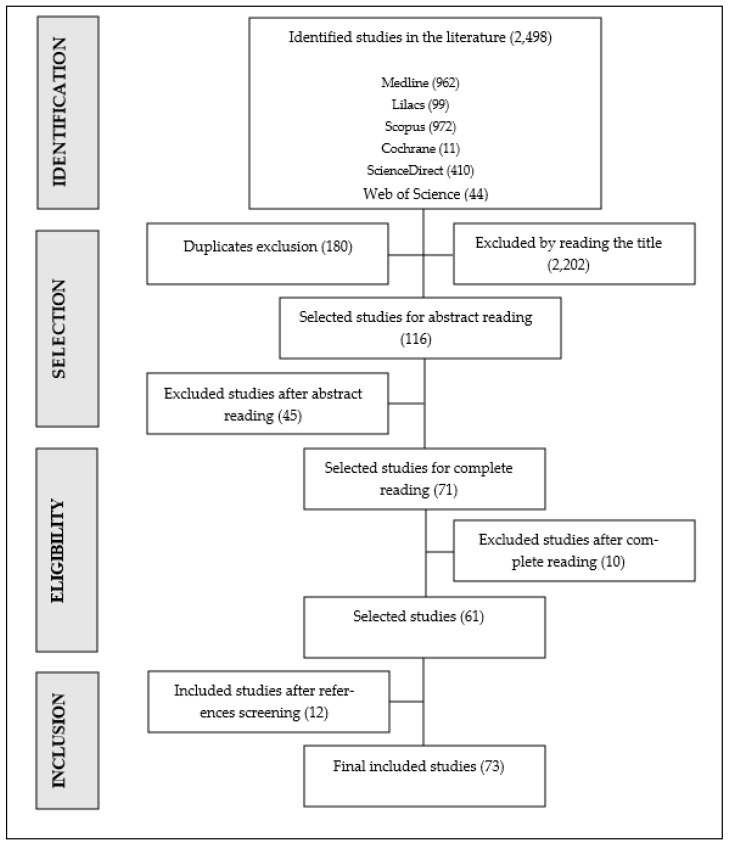
Flowchart of the study selection process. Source: adapted [17].

Phase I was performed by two reviewers independently. Eligibility criteria were applied for selection by titles and abstracts. Then, in phase II, also carried out by two independent reviewers, the full texts were analyzed, again applying the adopted eligibility criteria. Also, the authors evaluated the list of references from the included studies. In phase II, the exclusions were justified. At all stages, disagreements were resolved in a consensus meeting. Contacts were made with experts to identify whether any study was left out of the search, as described in Figure 1.

The review results are presented in a descriptive format, using tables to summarize data from the studies, following the JBI recommendations [18].

### 2.6. Summary of Results

The results were synthesized by qualitative analysis, with information presented in narrative, tabular, and/or graphic form. The studies were evaluated by identifying the used methodologies and the prevalence of the methods, approaches, criteria, and indicators reported from the proportions based on the number of included studies.

In addition, the information was synthesized and subdivided into ten groups: user profile; users’ health; handlers/workers; menu, food consumption and food health; hygienic–sanitary quality; assessment of (in) food and nutrition security, nutrition education, and the human right to adequate food; implementation, history, perceptions, senses, and meanings; physical–functional planning; rest intake; evaluation and monitoring.

## 3. Results

We found 2498 studies, of which 180 duplicates were excluded. After reading the titles, another 2202 articles were excluded. Of the 116 studies selected for abstract reading, 45 were excluded for not meeting the eligibility criteria. Among the 71 full-text reading studies, 10 did not correlate with the research objective, and 12 were included after reference analysis, totaling 73 studies in the scoping review. Figure 1 illustrates the selection process. 

Of the included studies, 57 had a quantitative approach, 14 had a qualitative approach, and 2 were mixed (quantitative/qualitative). Of the 73 studies, the main study design was cross-sectional (n = 60; 82.2%), followed by case studies (n = 10; 13.7%) (Table 1).

Most of the studies were conducted in Brazil (52), 9 in the United States, 5 in Peru, 2 in Mexico, 2 in Canada, 2 in Australia, and 1 in Argentina (Table 2). 

Regarding the type of evaluation, 20 studies evaluated the menu, food consumption, and food health; 17 Food and nutritional security, nutrition education, and the human right to adequate food; 13 the user’s profile; 12 the health of users; 12 the implantation, history, perceptions, senses, and meanings; 8 handlers/workers; 4 evaluation and monitoring; 3 hygienic–sanitary quality; 3 physical–functional planning; and 2 intake/rest/consumption (Table 3). 

A study was allocated to more than one category when it performed more than one type of evaluation. The description of the methods, criteria, and indicators used in evaluating CRs are presented in Table 4. 

## 4. Discussion

Community restaurants have been evaluated since the 1990s, initially seeking to describe the population that accessed these facilities, focusing on sociodemographic profiles and the reasons that led these users to frequent such spaces. After this initial period, other research aimed to evaluate the quality of the food served and its impact on the health of users and the situation of food insecurity. Studies have focused on evaluating community restaurant programs, shifting from looking at users to focusing on the program concerning achieving its objectives for society.

The main approach in the evaluations of the CR was the quantitative one, which is defined by the work carried out with variables expressed in the form of numerical data, which rigid resources and statistical techniques are used to classify and analyze, such as the percentage, mean, standard deviation, correlation coefficient, and regressions, among others. As they express greater precision and reliability, quantitative studies are more suitable for planning group actions, as their results are likely to be generalized, primarily when the surveyed samples faithfully represent the population from which they were taken. On the other hand, the qualitative approach seeks to understand specific complex phenomena of a social and cultural nature through descriptions, interpretations, and comparisons without considering their numerical aspects (mathematical and statistical rules) [85,86]. For this dichotomy to be overcome, some researchers seek mixed methods. The choice of quantitative, qualitative, or mixed studies is mainly based on the research questions and chosen variables. However, quantitative studies have been the most chosen option, not only to answer such research questions but mainly because such studies are used for the direct or indirect evaluation of food and nutritional security programs. Subsidies are offered when considering the maintenance of programs with indicators such as cost/benefits or compliance with the user profile to be covered by policies to overcome the population’s social vulnerability.

As for the study design, the cross-sectional design is the most predominant. Compared to other designs, this type of study is easily carried out, fast, economical, and very useful in public health. In addition, it offers a better cost/benefit ratio for planning and evaluating public programs, as already mentioned. This study design analyzes well-defined populations, with its fundamental characteristic being the measurement at a single moment [85,86]. However, such designs have limitations. In general, it is impossible to determine causality to ensure that confounding factors will be equally distributed between the groups, and there is no way to guarantee that they are not compromised by prevalence bias (cured and deceased people are excluded). Nevertheless, the sample’s internal groups may have very different sizes, resulting in a loss of statistical efficiency. Still, its strengths outweigh its limitations [87]. 

Another type of study that stood out was the case studies, characterized as studies of a well-defined entity such as a program, an institution, an educational system, a person, some people, or a social unit. This study design seeks to understand in depth how and why a particular situation, which is supposed to be unique in many aspects, leads to such behavior. Therefore, it seeks to discover what is more important and characteristic about this object from the participants’ point of view. It always intends to analyze the case objectively and pragmatically or present a global perspective of the event (case) [86,88]. 

Regarding the origin of the studies, Brazil, followed by the United States, gained greater prominence. This arises from the fact that Brazil currently has a well-structured program that offers meals, preferably to the low-income population, as part of the food assistance strategies integrated into the Brazilian federal government´s network of social inclusion and hunger-fighting policies [7,26,65,67]. The United States, due to its federalism model and regarding the preparation of the meals and the financing of these initiatives, presents state or municipal initiatives with the participation of the local government and volunteers. These initiatives are vital to the food security of the North American low-income community [5,89]. 

Observing the subdivisions adopted by this study, the evaluation of the served menu, food consumption, and dietary health were the most studied (20 studies). This fact comes from the researchers’ concern for evaluating whether these restaurants, even offering the meal for free or at subsidized prices (USD 0.20 to 1.02), produce a quality meal that promotes users’ health. To this end, they used dietary surveys through food history, which met the inclusion criterion of a frequency of meals no less than three times a week during the last six months. Obtaining data related to the users’ dietary information included the technique of retrospective dietary assessment (35%), application of food frequency (25%), the 24 h recall (30%), and/or direct weighing of food [90,91,92,93,94]. Retrospective methods included the 24 h recall, food frequency questionnaires, and food history. When applying the recording method, the food and drinks consumed in the last 24 h were quantified, describing the type of food and drink, size and/or volume, preparation method, and fractionation. The food frequency consisted of applying a list of foods in which the interviewee indicated how often each food was consumed in a given period. The direct weighing of food consisted of a prospective method carried out by weighing the food discounting the inedible parts and leftovers. The various methods for the quantitative assessment of food consumption provide information not only on the meals consumed in the CR but also on how they contribute to the user’s day and the coverage of nutritional needs. According to the Council of the Institute of Medicine, Food, and Nutrition, three consecutive days of assessment of food consumption is representative of an individual’s diet. It constitutes the gold standard for obtaining data on the food consumption of individuals and/or populations [93].

For the food consumption investigation, part of the questionnaire for surveillance risk and protective factors for chronic diseases by telephone survey (VIGITEL) was used. VIGITEL is a Brazilian survey carried out by the Ministry of Health, created to monitor risk and protective factors for chronic non-communicable diseases in all capitals of the Brazilian states and in the federal district. Data collection is carried out through telephone calls to interviewees, and the questionnaire is composed of sociodemographic and health variables, providing information on the population’s habits in relation to food, physical activity, smoking and the consumption of alcoholic beverages, and the existence of diseases such as diabetes, hypertension, and depression, among others [95], in addition to the offer of the total caloric value, the caloric density of the meal [40,96], regional food offer, the number of calories provided by dietary liquid protein [97], and macro and micronutrients [98,99], to verify if the daily supply of calories, protein, and nutrients is adequate. Although the methods used to determine consumption are highly different, the choice for a certain method was undoubtedly due to the cost/benefit ratio, not the precision. VIGITEL, for example, is an investigation carried out through telephone calls and, therefore, subject to biases inherent to the desirability and memory of individuals.

The nutritional composition of the meal (45% of the studies) was evaluated using technical preparation files [100], direct food weight, and food composition tables [101]. Technical preparation files consist of an operational management support instrument, which aims to survey costs, order preparation, and calculate the nutritional value of the preparation to be carried out. They help support menu planning. Meal suitability values were compared to requirements specified by the Food and Drug Administration standards [101], Centers for Disease Control and Prevention [96], Food and Agriculture Organization of the United Nations [102], and National Health Surveillance Agency [97]. The choice of these strategies that determine and evaluate consumption and adequacy were adopted, possibly because they can be replicated by subsequent studies and compared with national or international standards.

These studies aimed to assess the adequate supply of nutrients by restaurants. The studies indicated that meals served at the CR presented an energy density above the recommendations. However, these values can be justified because these restaurants have consumers that usually just have one meal a day, and this meal is consumed at the CR. Regarding the nutritional composition of the meals, the majority attended the nutritional needs for the lunch period, the most served meal at the CR. These studies aimed to assess the adequate supply of nutrients by restaurants. It is important to ensure the adequacy of the nutritional composition of menus, thus creating conditions for users to have a nutritionally healthy meal.

For the assessment of food safety/insecurity, in Brazil, there is the Brazilian Food Insecurity Scale [103]. In the United States, the Nutritional Knowledge Scale, Central Food Safety Module was used [104], and in Mexico, the Latin American Food Security Scale [105]. They all directly assess one of the dimensions of food and nutritional security in a population through the perception and experience of hunger. Hunger perception scales are direct indicators for assessing food insecurity but do not measure the nutritional dimension. Using the score obtained, the scales classify the assessed households into food security, mild food insecurity, moderate food insecurity, or severe food insecurity. They have become an assessment standard, as they can express food access and provide high reliability, reflecting the experience of life with food insecurity and hunger.

Nutrition education was developed through workshops, recreational activities, and focus groups to promote education for citizenship and create conditions for empowering the population regarding food and nutritional security issues and the right to food. This technique can be improved and transcribed to the local reality and culture. This allows for some instruments to be adjusted and validated in different countries and cultures, conducting studies resulting from applying such instruments, subject to comparison. In addition, a legal and institutional approach was used to evaluate the human right to adequate food, analyzing the limits and possibilities of the advances and preservation of the guarantee of the human right to adequate food [106]. These studies aimed at developing active citizenship among restaurant users who participate in social programs, in which society mobilizes and can demand from public authorities the fulfillment of the right to adequate food from a legal point of view.

Profile studies of restaurant users (13 studies) aimed to characterize frequent users to verify whether they serve the population with higher rates of social vulnerabilities. The main sociodemographic indicators used in these assessments were gender, age group, marital status, skin color, education, head of household, family composition, place of residence, housing condition, type of housing material, profession, per capita income, social class, formal contract, place of work, possession of goods, means of transportation used, motivation for having the meal, days on which meals are served, place where meals are served on weekends, beneficiaries of social programs, drug or alcoholic beverage users, practice of physical activity, and the presence of chronic diseases. In Brazil, most studies used the Brazilian Institute of Public Opinion instrument as their basis, which is a pioneering study on the evaluation of user profiles [107]. Carrying out this type of analysis enables a more detailed diagnosis of each region’s socioeconomic inequalities and specificities, thus facilitating the planning and execution of corrective actions consistent with the local reality. Majorly, CR users are low-income, non-white race, and have little educational background. Therefore, CRs follow their goals to offer meals to the most vulnerable population. Food access to this population is essential since low-income people eat less or no food, and frequently, do not have resources to buy it.

In the evaluations of the users’ health (12 studies), the studies aimed at evaluating whether the food served by the CR presented a connection with users’ diseases such as metabolic syndrome, cardiovascular diseases, chronic non-transmissible diseases, anemia, total cholesterol, triglycerides, high-density lipoproteins (HDL), low-density lipoprotein (LDL), and very low high-density lipoprotein (VLDL) [96,108,109,110] using methods already scientifically established by the WHO. Dyslipidemia, anemia, and blood glucose were measured in a fasting blood sample, and to determine glucose, total cholesterol, and triglycerides, enzymatic colorimetric methods were used. HDL was determined using the low- and very-low-density lipoprotein precipitation method using the cholesterol oxidase/peroxidase enzyme system with colorimetry. Blood pressure was measured twice with a properly calibrated aneroid sphygmomanometer. The concern in evaluating users’ health arises from the low cost of the food, making it possible to have concerns about the quality of the final product served to the consumer. The recommendations of the World Health Organization and the International Diabetes Federation were used to assess nutritional status (41.6% of the studies). The studies evaluated the user’s health to verify their current state of health and whether the food served by the restaurants had promoted the improvement of their health or the onset of diseases [98,108,110,111]. The CR’s primary objective is to serve low-cost food that is nutritionally healthy and does not harm the health of its guests.

Body mass index (BMI) and waist and abdominal circumference were also evaluated and correlated with the onset of health problems. To obtain the body mass index, the ratio of weight in kilograms to the square of height in meters (kg/m^2^) was calculated, while circumference measurements were obtained using a measuring tape. This type of assessment seeks to recognize the users’ dietary needs so that it is possible to intervene appropriately for health maintenance or recovery. The assessment of nutritional status through BMI is a good indicator of the accumulation of adipose tissue due to excess energy, and it is equally reliable for both genders and different ages. Other methods that could be used in this type of evaluation are densitometry and bioimpedance, which are quick, as they are performed in up to 12 min and do not require preparation. However, among the disadvantages are the high cost, the use of radiation during the evaluation, and the difficulty in transporting the equipment to different locations [112].

To evaluate the implementation, history, perceptions, senses, and meanings, the authors resorted to a sociohistorical analysis (41.6% of the 12 studies) [113,114], bibliographic research, documentary, and field research, and direct observation [115,116]. They created focus groups with managers, handlers, and users, seeking to understand each person´s life story and their perceptions about these food and nutrition establishments, which aim to provide access to food and fight hunger. However, this investigation method may not be as efficient in terms of the coverage of a specific topic compared to individual interviews because there is little depth on the subject. There is a possibility that members may not honestly express their personal opinions, especially if their ideas differ from those of other members [117]. According to Sordini [118], for the users of these spaces, the main perception is that the practices, from preparing food to eating the meals served by the CR, are based on love, trust, and hope as a possibility of meeting with the others, and it implies a standard view of possible, desirable, and shared horizons of action.

Studies on the evaluation of handlers/workers used all professionals who worked in different types of restaurants (administrators/managers, nutritionists, administrative assistants, cashiers, stock helpers, chefs, cooks, kitchen helpers, butchers, butlers, and general service assistants). They sought to characterize this population [118,119,120,121] and assess health and work conditions through the incorporation of scales used and validated in national [122,123] and international surveys [19,96,108,109,110,111,121,124]. Occupational physical exertion was classified according to the worker’s perception and followed the recommendations of Andersen, Izquierdo, and Sundstrup (2017) [122].

Occupational psychosocial characteristics were studied using the Job Content Questionnaire (JCQ), a validated and self-administered instrument designed to measure workers’ social and psychological characteristics. It is often used for the analysis of micro-level job characteristics, such as assessing the relative risks of individual exposures to different work settings, to predict the development of work-related illnesses, psychological distress, coronary heart disease, musculoskeletal diseases, and reproductive disorders [121]. The food and nutritional security level of workers/handlers was also evaluated using the food insecurity scales [103,104,105]. A questionnaire based on current Brazilian legislation on good handling practices (National Health Surveillance Agency) was applied to assess Brazilian food handlers’ knowledge and self-reported practice in two studies [123,125]. These studies served as a basis for revealing the characteristics of workers whose well-being is reflected in their health and their daily work practices.

Within the scope of the evaluation and monitoring studies, the construction of the proposal of the theoretical–logical model of Brazilian community restaurants was carried out [55] as a representation of the program and its movements and relationships, translating into theoretical and practical propositions for the evaluated object. A proposal for an assessment matrix was also developed [56] containing the restaurants’ dimensions, sub-dimensions, and evaluation indicators with their respective justifications. To build the theoretical–logical model and matrix, an in-depth literature review and consensus workshops were carried out, totaling 12 h, using the traditional committee technique. The matrix was evaluated by experts external to the research group with experience in CR implementation and management. In addition, the criteria of efficiency and effectiveness were used to create a value judgment of CRs [14,126]. To evaluate the effectiveness of the CR program, the proportion of coverage of the “target audience,” defined within the scope of the CR program, was estimated. Access to food was considered adequate effectiveness when the CR, within their possibilities, served meals to 50% to 70% of users considered as the program’s “target audience”. Data envelopment analysis (DEA) was used as the most appropriate methodology for the efficiency of public spending. For this purpose, the software MaxDEA version 12.0 for data envelopment analysis was used. For the evaluation and monitoring of public policies and/or instruments, the authors resorted to evaluation techniques according to the criteria of effectiveness and efficiency, which are guidelines for the planning and improvement of programs in public management [113,127,128].

The assessment of the hygienic–sanitary quality was verified using the sanitary inspection script based on the Resolution RDC nº 216/2004 of the National Health Surveillance Agency [123], from the checklist of Manual de buenas practicas de manipulación de alimentos para restaurantes y servicios afines [129] and the Official Mexican Standard NOM-093-SSA1-1994 de Practicas de hijiene y sanidad en la preparación de alimentos que se ofrecen en establecimientos Àjos [130]. They all aim to help traders and handlers prepare, store, and sell food appropriately, hygienically, and safely [123]. The evaluations were aimed at verifying the quality of the hygienic–sanitary conditions and identifying non-conformities that could interfere with the quality of the served meals, calculating the adequacy percentage of the hygienic–sanitary conditions [131,132]. These studies used standards and norms from the national legislation of each country. The main evaluated items were buildings, installations, furniture, and fixtures; the hygiene of facilities, equipment, furniture, and accessories; the integrated control of vectors and urban pests; water supply; waste management; handlers; raw materials, ingredients, and packaging; food preparation; the storage and transport of prepared foods; exposure to the consumption of prepared foods; documentation and records; standard operating procedures; and, finally, responsibility. For application in other countries, it is necessary to follow the guidelines and checklists approved by the national health surveillance agencies. In this sense, such instruments offer the possibility of determining which conditions are considered ideal for producing meals and which points require correction to obtain the ideal conditions. Furthermore, it is possible to analyze the conditions of restaurants in different countries after the required adaptations.

The physical–functional planning studies aimed to assist in the implementation of CRs. To this end, a list describing the equipment, utensils, and consumables by sector and their respective quantities was drawn up. It also describes the average cost of meals and base menu planning for five days (Monday to Friday). The analysis of the quantity of human resources necessary for the establishment’s operation was used as a basis for the calculations, using several parameters described by Teixeira et al. (2007) [83]. The organization chart of the staff was prepared, describing the positions and functions. Furthermore, when evaluating the installation of CRs, aspects related to location, zoning, sectors, and environment were observed based on the roadmap for the implementation of community restaurants published by the Ministry of Social Development and Fight against Hunger [133]. The studies in this subdivision are Brazilian and followed the federal government’s rules. The other studies did not assess physical–functional planning.

To determine the intake (consumption) of meals, the studies used the proportion between the food returned by users and the quantity of food distributed. By performing the calculation, the formula proposed by Vaz (2006) [84] was used to obtain the food waste index and was adopted in the evaluation of CRs. Thus, it was considered as a synonym of poor quality when the indexes were above the recommended percentages, which can be avoided through planning. This type of evaluation’s main objective is to promote diners’ awareness and minimize food waste. It should be noted that only two studies assessed this issue, one in Brazil and one in the United States. In both cases, the assessment of food losses was carried out using the same formula. It is crucial to raise awareness of the need to reduce waste as one of the strategies for the sustainability of restaurants. Food waste is one of the sustainability assessment pillars in restaurants [134]. In this view, sustainable actions must be developed within the most diverse stages involving the meal production process, thus contributing to increasing the quality of the service provided and sustainability [135].

Systematizing and disseminating findings regarding CRs assessments can contribute to identifying the most prevalent state practices and policies, research gaps that may indicate which methods and tools should be created and consolidated, and how research in this area is being conducted. In this way, the scoping review can help the reviewer examine emerging evidence when the existing scientific production is recent and/or incipient and examine how research is being conducted in already consolidated areas that can generate knowledge with the potential to guide decisions and actions in public policy.

This study presents limitations inherent to systematic reviews, or not, such as, in some cases, not all studies are included in the main databases, and it does not propose, in the case of the scoping review, to evaluate the quality of the included studies. At the same time, materials and research that are not published in scientific journals and databases, such as government documents, are not included and could have provided more information about CRs.

The search used eight databases, allowing a greater number of studies in the evaluated area to be found. Accordingly, the review includes a variety of studies published since the 1990s on evaluations carried out on CRs, which allowed the identification of different evaluative approaches using quantitative, qualitative, and mixed methods, in addition to presenting remarkable experiences in several countries.

## 5. Conclusions

Seventy-three studies were published since the 1990s in different countries, mainly in Brazil. The evaluative approaches dealt with the menu, food consumption, food health, food security and/or insecurity, nutrition education, the human right to adequate food; user profile and health; implantation, history, perceptions, senses, and meanings; handlers/workers; hygienic–sanitary quality; evaluation and monitoring; physical–functional planning; and rest intake. The results increase the comprehension of evaluation methods performed at the CR. They provide details on methods, approaches, criteria, and indicators that can be used and/or adapted in future evaluations. The results also describe the area’s academic production panorama.

In this scenario, progress on the methods, criteria, and indicators used in CRs is necessary to better investigate the nutritional and food security framework. The evaluations performed in these establishments must be strategies inherent to the programs, being fundamental for their qualification and goal achievement.

Furthermore, the scoping review is appropriate to examine studies for decision making in the theoretical–methodological field, from mapping theories to methodologies that should inform researchers. Systematizing and disseminating findings fulfill the objective of contributing to practices and policies.

## Figures and Tables

**Table 1:** Epidemiological approach and types of studies included in the scoping review; Brazil, 2023.

**N**	**Approach**	**Studies**
57	Quantitative	Adams; Chirinos [1] (2018); Aguiar; Valente; Fonseca [19] (2010); Amorim; Silva; Gomes [20] (2007); Araújo; Almeida; Bastos [21] (2007); Assunção et al. [22] (2017); Bento et al. [23] (2016); Boas et al. [24] (2021); Botelho; Akutsu; Zandonadi [25] (2019); Branquinho et al. [26] (2015); Canonico; Pagamunici; Ruiz [27] (2014); Caro et al. [28] (2018); Carrijo et al. [29] (2018); Costa et al. [30] (2022); Costa; Horta; Ramos [31] (2019); Darley et al. [32] (2021); Duarte; Botelho; Akutsu [33] (2019); Eppich; Fernandez [5] (2004); Falcão; Aguiar; Fonseca [34] (2015); Fano; Tyminski; Flynn [35] (2004); Fideles et al. [36] (2020); Fideles et al. [37] (2021); Fideles et al. [38] (2022); Freedman; Bartoli [39] (2013); Ginani et al. [40] (2018); Gobato; Panigassi; Villalba [41] (2010); Godoy et al. [7] (2014); Godoy et al. [42] (2017); Gomes; Pereira; Abreu [43] (2018); Gonçalves; Campos; Sarti [44] (2011); Hidalgo; Chuquinaupa; Luna [45] (2009); Kirchheim; Garcia; Baratto [46] (2021); Koh; Bharel; Henderson [47] (2016); Lee et al. [48] (2010); Lima et al. [49] (2020); Machado et al. [50] (2014); Mello et al. [51] (2013); Minuzzi et al. [52] (2018); Moraes, Godoy; Oliveira [53] (2015); Mousa; Freeland-Graves [54] (2019); Oliveira et al. [55] (2019); Oliveira et al. [56] (2020); Oyhenart et al. [8] (2007); Poluha; Motta; Gatti [57] (2016); Portella; Basso; Medina [58] (2013); Rausschenbach et al. [59] (1990); Ribeiro et al. [60] (2017); Rosenblum et al. [61] (2005); Silva; Pedelhes; Costa [62] (2018); Sisson; Lown [63] (2011); Sobrinho et al. [64] (2014); Sousa et al. [65] (2019); Sousa et al. [66] (2020); Sousa et al. [67] (2021); Souza et al. [68] (2014); Souza; Azevedo; Seabra [69] (2018); Souza; Marín-León [70] (2013); Zanette et al. [71] (2021).
14	Qualitative	Andrade [2] (2016); Araújo et al. [72] (2015); Braun; Costa [73] (2019); Buttorff et al. [74] (2015); Dachner et al. [4] (2009); Drinot [75] (2005); Furber et al. [6] (2010); Hosseini [76] (2017); kayman et al. [77] (2005); Nunes; Andrade [78] (2013); Padrão; Aguiar [79] (2018); Ramírez; Moreira; Oliveira [80] (2016); Ramos et al. [81] (2020); Souza; Belik [9] (2012).
2	Mixed	Balam-Gómez et al. [3] (2013); Creed-Kanashiro et al. [82] (2013).
**N**	**Type**	**Studies**
60	Cross-sectional	Adams; Chirinos [1] (2018); Aguiar; Valente; Fonseca [19] (2010); Araújo; Almeida; Bastos [21] (2007); Assunção et al. [22] (2017); Balam-Gómez et al. [3] (2013); Bento et al. [23] (2016); Boas et al. [24] (2021); Botelho; Akutsu; Zandonadi [25] (2019); Branquinho et al. [26] (2015); Canonico; Pagamunici; Ruiz [27] (2014); Caro et al. [28] (2018); Carrijo et al. [29] (2018); Costa et al. [30] (2022); Costa; Horta; Ramos [31] (2019); Creed-Kanashiro et al. [82] (2013); Darley et al. [32] (2021); Duarte; Botelho; Akutsu [33] (2019); Eppich; Fernandez [5] (2004); Falcão; Aguiar; Fonseca [34] (2015); Fano; Tyminski; Flynn [35] (2004); Fideles et al. [36] (2020); Fideles et al. [37] (2021); Fideles et al. [38] (2022); Freedman; Bartoli [39] (2013); Furber et al. [6] (2010); Ginani et al. [40] (2018); Gobato; Panigassi; Villalba [41] (2010); Godoy et al. [7] (2014); Godoy et al. [42] (2017); Gomes; Pereira; Abreu [43] (2018); Gonçalves; Campos; Sarti [44] (2011); Hidalgo; Chuquinaupa; Luna [45] (2009); Kirchheim; Garcia; Baratto [46] (2021); Koh; Bharel; Henderson [47] (2016); Lee et al. [48] (2010); Lima et al. [49] (2020); Machado et al. [50] (2014); Mello et al. [51] (2013); Minuzzi et al. [52] (2018); Moraes, Godoy; Oliveira [53] (2015); Mousa; Freeland-Graves [54] (2019); Oliveira et al. [55] (2019); Oliveira et al. [56] (2020); Oyhenart et al. [8] (2007); Poluha; Motta; Gatti [57] (2016); Portella; Basso; Medina [58] (2013); Rausschenbach et al. [59] (1990); Ribeiro et al. [60] (2017); Rosenblum et al. [61] (2005); Silva; Pedelhes; Costa [62] (2018); Sisson; Lown [63] (2011); Sobrinho et al. [64] (2014); Sousa et al. [65] (2019); Sousa et al. [66] (2020); Sousa et al. [67] (2021); Souza et al. [68] (2014); Souza; Azevedo; Seabra [69] (2018); Souza; Marín-León [70] (2013); Zanette et al. [71](2021).
10	Case Studies	Araújo et al. [72] (2015); Balam-Gómez et al. [3] (2013); Braun; Costa [73] (2019); Buttorff et al. [74] (2015); Creed-Kanashiro et al. [82] (2013); Dachner et al. [4] (2009); Hosseini [76] (2017); Nunes; Andrade [78] (2013); Ramírez; Moreira; Oliveira [80] (2016); Ramos et al. [81] (2020).
4	Documental	Andrade [2] (2016); Drinot [75] (2005); Padrão; Aguiar [79] (2018); Souza; Belik [9] (2012).
1	Cohort	Amorim; Silva; Gomes [20] (2007).
1	Intervention	Kayman et al. [77] (2005).

**Table 2:** Studies included in the scoping review according to authorship/year of publication, title, country of origin, and publication type; Brazil, 2023.

Study	Authors/Year	Title	Country
1	Adams; Chirinos [1] (2018).	Prevalence of risk factors for metabolic syndrome and its components in users of soup kitchens in a district of Lima, Peru	Peru
2	Aguiar; Valente; Fonseca [19] (2010).	Socio-demographic, labor, and health description of workers in the food services sector of community restaurants in the state of Rio de Janeiro	Brazil
3	Amorim; Silva; Gomes [20] (2007).	Social Investment and Users’ Profile of the First Community Restaurant in Belo Horizonte-MG.	Brazil
4	Andrade [2] (2016).	“Abundant, healthy and cheap food”: Popular restaurants in Santiago (1936–1942).	Chile
5	Araújo et al. [72] (2015).	Community Restaurant Program: an alternative to promote the human right to adequate food?	Brazil
6	Araújo; Almeida; Bastos [21] (2007).	Food and Nutritional Aspects of Users of “Restaurante Popular Mesa do Povo”.	Brazil
7	Assunção et al., [22] (2017).	Socioeconomic, demographic, and food profile of users of the community restaurant in Juiz de Fora, MG.	Brazil
8	Balam-Gómez et al. [3] (2013).	Evaluation of community kitchens in Tizimín, Yucatán, Mexico: perceptions and proposals of staff and beneficiaries.	Mexico
9	Bento et al. [23] (2016).	Factors associated with the eating behavior phases of users of community restaurants in Belo Horizonte/MG-Brazil.	Brazil
10	Boas et al. [24] (2021).	Access to regional food in Brazilian community restaurants to strengthen the sustainability of local food systems.	Brazil
11	Botelho; Akutsu; Zandonadi [25] (2019).	Low-Income Population Sugar (Sucrose) Intake: A Cross-Sectional Study among Adults Assisted by a Brazilian Food Assistance Program.	Brazil
12	Branquinho et al. [26] (2015).	Health and sociodemographic profile of the clientele of restaurants linked to the Brazilian social program.	Brazil
13	Braun; Costa [73] (2019).	Impact of community restaurants on health and social development of users: the case of Toledo (PR).	Brazil
14	Buttorff et al. [74] (2015).	Evaluating consumer preferences for healthy eating from Community Kitchens in low-income urban areas: a discrete choice experiment of *Comedores Populares* in Peru.	Peru
15	Canonico; Pagamunici; Ruiz [27] (2014).	Evaluation of leftovers and rest-intake in a community restaurant in the city of Maringa-PR.	Brazil
16	Caro et al. [28] (2018).	Level of Food Security in beneficiaries of Community Kitchens of the National Crusade against Hunger program (Mexico).	Mexico
17	Carrijo et al. [29] (2018).	Is What Low-Income Brazilians Are Eating in Popular Restaurants Contributing to Promote Their Health?	Brazil
18	Costa et al. [30] (2022).	Obesity among government-backed economy restaurant workers in Belo Horizonte, Brazil.	Brazil
19	Costa; Horta; Ramos [31] (2019).	Food insecurity and overweight among government-backed economy restaurant Workers.	Brazil
20	Creed-Kanashiro et al. [82] (2013).	Formative research to develop a nutrition education intervention to improve dietary iron intake among women and adolescent girls through community kitchens in Lima, Peru.	Peru
21	Dachner et al. [4] (2009).	An ethnographic study of meal programs for homeless and under-housed individuals in Toronto.	Canada
22	Darley et al. [32] (2021).	Nutritional profile of users of a community restaurant.	Brazil
23	Drinot [75] (2005).	Food, Race and Working-Class Identity: Popular Restaurants and Populism in 1930s Peru.	Peru
24	Duarte; Botelho; Akutsu [33] (2019).	Regional food consumption in the Northeast of Brazil by the low-income population.	Brazil
25	Eppich; Fernandez [5] (2004).	Study finds Chapel Hill, NC, soup kitchen serves nutritious meals.	United States
26	Falcão; Aguiar; Fonseca [34] (2015).	Association of socioeconomic, labor, and health variables related to Food Insecurity in Popular Restaurants’ workers in Rio de Janeiro.	Brazil
27	Fano; Tyminski; Flynn [35] (2004).	Evaluation of a collective kitchens program using the population health promotion.	Canada
28	Fideles et al. [36] (2020).	Brazilian community restaurants’ low-income food handlers: Association between the nutritional status and the presence of non-communicable chronic diseases.	Brazil
29	Fideles et al. [37] (2021).	Food Insecurity among Low-Income Food Handlers: A Nationwide Study in Brazilian Community Restaurants.	Brazil
30	Fideles et al. [38] (2022).	Brazilian Food Handlers’ Years of Work in the Foodservice and Excess Weight: A Nationwide Cross-Sectional Study.	Brazil
31	Freedman; Bartoli [39] (2013).	Food intake patterns and plate waste among community meal center guests show room for improvement.	United States
32	Furber et al. [6] (2010).	The role of a community kitchen for clients in a socio-economically disadvantaged neighborhood.	Australia
33	Ginani et al. [40] (2018).	What is offered by public foodservices for the low-income population in Brazil is adequate to health promotion regarding energy density.	Brazil
34	Gobato; Panigassi; Villalba [41] (2010).	User profile identification of a community restaurant in the city of Campinas.	Brazil
35	Godoy et al. [42] (2017).	Food insecurity and nutritional status of individuals in a socially vulnerable situation in Brazil.	Brazil
36	Godoy et al. [7] (2014).	Profile and situation of food insecurity among users of Community Restaurants in Brazil.	Brazil
37	Gomes; Pereira; Abreu [43] (2018).	Factors associated with self-rated health among elderly users of community restaurants in Belo Horizonte.	Brazil
38	Gonçalves; Campos; Sarti [44] (2011).	Public food safety policies in Brazil: an analysis of the Community Restaurants Program.	Brazil
39	Hidalgo; Chuquinaupa; Luna [45] (2009).	Risk factors for metabolic syndrome in female members of soup kitchens in Cercado de Lima.	Peru
40	Hosseini [76] (2017).	Food insecurity and the use of soup kitchens among suburban elderly women in two counties in Pennsylvania.	United States
41	kayman et al. [77] (2005).	“A Port in a Storm”: Client Perceptions of Substance Abuse Treatment Outreach in a Soup Kitchen.	United States
42	Kirchheim; Garcia; Baratto [46] (2021).	Implementation of a community restaurant in a municipality in the interior of Paraná: contributions to the physical and functional planning of the place.	Brazil
43	Koh; Bharel; Henderson [47] (2016).	Nutrition for homeless populations: shelters and soup kitchens as opportunities for intervention.	United States
44	Lee et al. [48] (2010).	Process evaluation of community kitchens: results from two Victorian local government areas.	Australia
45	Lima et al. [49] (2020).	Anthropometric and Chronic Noncommunicable Diseases Profile of Elderly People Who Visit Community Restaurants in the Interior of Rio Grande do Norte-RN.	Brazil
46	Machado et al. [50] (2014).	Factors associated with overweight in adult users of community restaurants in Belo Horizonte, Brazil.	Brazil
47	Mello et al. [51] (2013).	Physical-functional structure of community restaurants in the state of Rio de Janeiro: influence on hygienic-sanitary conditions.	Brazil
48	Minuzzi et al. [52] (2018).	Nutritional status and sociodemographic profile of users of community restaurants in Caxias do Sul.	Brazil
49	Moraes, Godoy; Oliveira [53] (2015).	Diagnosis of Food Insecurity and the Nutritional Status of users of community restaurants in the Northeast and South of Brazil.	Brazil
50	Mousa; Freeland-Graves [54] (2019).	Food security of food recipients of a food pantry and soup kitchen.	United States
51	Nunes; Andrade [78] (2013).	The meaning of the Community Restaurant of Maracanaú as a public food and nutrition facility for its users.	Brazil
52	Oliveira et al. [55] (2019).	Government-subsidized restaurants as promoters of the realization of the human right to adequate food: proposal of an evaluation model.	Brazil
53	Oliveira et al. [56] (2020).	Government-subsidized restaurants in Brazil: an evaluation within the framework of food and nutrition security.	Brazil
54	Oyhenart et al. [8] (2007).	Nutritional status and body composition of poor children residing in peripheral neighborhoods of La Plata, Argentina.	Argentina
55	Padrão; Aguiar [79] (2018).	Community Restaurant: he social policy in question.	Brazil
56	Poluha; Motta; Gatti [57] (2016).	Nutritional evaluation of meals and physical structure analysis in a community restaurant in Sorocaba-SP.	Brazil
57	Portella; Basso; Medina [58] (2013).	User profile of Community Restaurant in the city of Santa Maria-RS.	Brazil
58	Ramírez; Moreira; Oliveira [80] (2016).	Menu evaluation and identification of functional foods: qualitative study of a community restaurant in Araraquara, São Paulo, Brazil.	Brazil
59	Ramos et al. [81] (2020).	Evaluation of the quality of meals served in a community restaurant.	Brazil
60	Rausschenbach et al. [59] (1990).	Dependency on soup kitchens in urban areas of New York State.	United States
61	Ribeiro et al. [60] (2017).	Socioeconomic characterization, nutritional status and prevalence of food insecurity in elderly users of community restaurants in a municipality in northeastern Brazil.	Brazil
62	Rosenblum et al. [61] (2005).	Motivationally enhanced group counseling for substance users in a soup kitchen: A randomized clinical trial.	United States
63	Silva; Pedelhes; Costa [62] (2018).	The efficiency of spending on food security programs: The case of community restaurants in the Federal District.	Brazil
64	Sisson; Lown [63] (2011).	Do soup kitchen meals contribute to suboptimal nutrient intake & obesity in the homeless population?	United States
65	Sobrinho et al. [64] (2014).	Determining factors of food and nutrition insecurity: a study carried out in Popular Restaurants in Belo Horizonte, Minas Gerais, Brazil.	Brazil
66	Sousa et al. [65] (2019).	Nutritional quality of breakfast consumed by the low-income population in Brazil: A nationwide cross-sectional survey.	Brazil
67	Sousa et al. [66] (2020).	Breakfast characterization and consumption by low-income Brazilians: Food identity and regional food.	Brazil
68	Sousa et al. [67] (2021).	Evaluation of the effectiveness of Brazilian community restaurants for the dimension of low-income people access to food.	Brazil
69	Souza et al. [68] (2014).	Profile of Community Restaurant users in the central region of the state of Rio Grande do Sul.	Brazil
70	Souza; Azevedo; Seabra [69] (2018).	Food safety in Brazilian popular public restaurants: Food handlers’ knowledge and practices.	Brazil
71	Souza; Belik [9] (2012).	Food policy planning: an analysis based on the cases of Mexico, Brazil and Peru.	Brazil
72	Souza; Marín-León [70] (2013).	Food insecurity among the elderly: Cross-sectional study with soup kitchen users.	Brazil
73	Zanette et al. [71] (2021).	Systemic arterial hypertension and associated factors in users of the popular restaurant in Caxias do Sul-RS.	Brazil

Adapted from the JBI Model source of evidence details, characteristics, and result extraction instrument [18].

**Table 3:** Study division according to the evaluation area; Brazil, 2023.

Evaluation Area	N	Studies
Menu, food consumption, and food health	20	Araújo; Almeida; Bastos [21] (2007); Assunção et al. [22] (2017); Bento et al. [23] (2016); Boas et al. [24] (2021); Botelho; Akutsu; Zandonadi [25] (2019); Braun; Costa [73] (2019); Buttorff et al. [74] (2015); Carrijo et al. [29] (2018); Duarte; Botelho; Akutsu [33] (2019); Eppich; Fernandez [5] (2004); Ginani et al. [40] (2018); Mousa; Freeland-Graves [54] (2019); Poluha; Motta; Gatti [57] (2016); Ramírez; Moreira; Oliveira [80] (2016); Ramos et al. [81] (2020); Sisson; Lown [63] (2011); Sousa et al. [65] (2019); Sousa et al. [66] (2020); Sousa et al. [67] (2021); Zanette et al. [71] (2021).
Assessment of food and nutrition security, nutrition education and the human right to adequate Food	17	Araújo et al. [72] (2015); Caro et al. [28] (2018); Costa; Horta; Ramos [31] (2019); Falcão; Aguiar; Fonseca [34] (2015); Fano; Tyminski; Flynn [35] (2004); Godoy et al. [7] (2014); Godoy et al. [42] (2017); Gomes; Pereira; Abreu [43] (2018); Gonçalves; Campos; Sarti [44] (2011); Koh; Bharel; Henderson [47] (2016); Moraes, Godoy; Oliveira [53] (2015); Mousa; Freeland-Graves [54] (2019); Oliveira et al. [55] (2019); Oliveira et al. [56] (2020); Ribeiro et al. [60] (2017); Sobrinho et al. [64] (2014); Souza; Marín-León [70] (2013).
User Profile	13	Amorim; Silva; Gomes [20] (2007); Assunção et al. [22] (2017); Bento et al. [23] (2016); Darley et al. [32] (2021); Gobato; Panigassi; Villalba [41] (2010); Godoy et al. [7] (2014); Gomes; Pereira; Abreu [43] (2018); Gonçalves; Campos; Sarti [44] (2011); Minuzzi et al. [52] (2018); Portella; Basso; Medina [58] (2013); Ribeiro et al. [60] (2017); Sobrinho et al. [64] (2014); Souza et al. [68] (2014).
User Health	12	Adams; Chirinos [1] (2018); Creed-Kanashiro et al. [82] (2013); Darley et al. [32] (2021); Fano; Tyminski; Flynn [35] (2004); Hidalgo; Chuquinaupa; Luna [45] (2009); Lima et al. [49] (2020); Machado et al. [50] (2014); Minuzzi et al. [52] (2018); Oyhenart et al. [8] (2007); Rosenblum et al. [61] (2005); Souza; Marín-León [70] (2013); Zanette et al. [71] (2021).
Implementation, history, perceptions, senses, and meanings	12	Andrade [2] (2016); Balam-Gómez et al. [3] (2013); Buttorff et al. [74] (2015); Dachner et al. [4] (2009); Drinot [75] (2005); Furber et al. [6] (2010); Hosseini [76] (2017); kayman et al. [77] (2005); Lee et al. [48] (2010); Padrão; Aguiar [79] (2018); Rausschenbach et al. [59] (1990); Souza; Belik [9] (2012).
Handlers/workers	8	Aguiar; Valente; Fonseca [19] (2010); Costa et al. [30] (2022); Costa; Horta; Ramos [31] (2019); Falcão; Aguiar; Fonseca [19] (2015); Fideles et al. [36] (2020); Fideles et al. [37] (2021); Fideles et al. [38] (2022); Souza; Azevedo; Seabra [69] (2018).
Evaluation and monitoring	4	Oliveira et al. [55] (2019); Oliveira et al. [56] (2020); Silva; Pedelhes; Costa [62] (2018); Sousa et al. [67] (2021).
Hygienic–sanitary quality	3	Balam-Gómez et al. [3] (2013); Mello et al. [51] (2013); Souza; Azevedo; Seabra [69] (2018).
Physical–functional planning	3	Kirchheim; Garcia; Baratto [46] (2021); Mello et al. [51] (2013); Poluha; Motta; Gatti [57] (2016).
Rest intake	2	Canonico; Pagamunici; Ruiz [27] (2014); Freedman; Bartoli [39] (2013).

**Table 4:** The methods, criteria, and indicators used to evaluate community restaurants linked to government food and nutritional safety programs; Brazil, 2023.

Evaluation Area	N	%	Methods, Criteria, and Indicators
Menu, food consumption, food health	20		
	7	35.0	Retrospective food survey
	5	25.0	Food frequency questionnaire
	6	30.0	24H Recall (3 days)
	3	15.0	Weighing and direct observation of the consumer’s meal (3 times)
	1	5.0	Food Questionnaire of the Surveillance of Chronic Diseases by Telephone Survey (VIGITEL)
	3	15.0	Menu food daily offer questionnaire
	1	5.0	Diet Quality Index (DQI)
	1	5.0	Healthy Eating Index-2010
	1	5.0	Caloric Adequacy by the Adapted Food Pyramid
	9	45.0	Evaluation of the menu’s nutritional composition
	2	10.0	Qualitative Analysis of Menu Preparations (AQPC)
	1	5.0	Research Questionnaire on Eating Habits
	2	10.0	Evaluation of user satisfaction using a 7-point Likert scale
Assessment of food and nutrition security, nutrition education, and the human right to adequate food	17		
	1	5.9	Latin American Food Safety Scale—ELCSA
	9	52.8	Brazilian Food Insecurity Scale (EBIA)
	1	5.9	Questionnaire about new knowledge learned under the Program (Likert scales)
	1	5.9	Questionnaire about behavior since they use a community kitchen (Likert scales)
	1	5.9	Questionnaire of current practices, barriers, and ideas to improve the nutrition of homeless people
	1	5.9	Nutritional Knowledge Scale, Central Food Safety Module (CFSM)
	1	5.9	Normative evaluation
	1	5.9	Assessment matrix of two community restaurants in Brazil
	1	5.9	Questionnaire on the understanding of the Human Right to Adequate Food and the Community Restaurant Program
User Profile	13		
	13	100.0	Sociodemographic and socioeconomic questionnaire (sex, age group, marital status, degree of education, place of residence, possession of a fixed residence, profession, place of work, personal and family income, possession of assigned portfolio, the composition of family income, average transport used to go to restaurants, frequency use of the restaurant, type of catering (breakfast, lunch, and dinner), motivation and evaluation of the services), concern with the quality of the foods they eat, participant’s perception of the quality of their food, a beneficiary of social program
User Health	12		
	3	25.0	International Federation of Diabetes Criteria (abdominal circumference, glycemia, glucose, total cholesterol, triglycerides, HDL, LDL, VLDL)
	3	25.0	Blood pressure measurement
	5	41.6	Nutritional status
	1	8.3	Nutritional status for children (height for age, weight for age and weight for height)
	1	8.3	Verification of the Body Mass Index for the elderly (BMI)
	1	8.3	WHO Questionnaire for the evaluation of the practice of physical activity (18 to 64 years old)
	2	16.6	The practice of physical activity (does not perform, ≤3 times a week and >3 times a week)
	1	8.3	Anemia
	1	8.3	Consumption of alcoholic beverages (frequency per week or day)
	3	25.0	Waist circumference measurement
	1	8.3	Measurement of the circumference of the hip
	1	8.3	Measurement of abdominal circumference
	1	8.3	Brachial circumference measurement
	1	8.3	Subcutaneous triciptal
	1	8.3	Biological tests for HIV and drugs (hair of scalp)
	1	8.3	Cognitive-behavioral therapy (CBT)
	1	8.3	Assessment of depression (measured with the version of the other items of the CES-D)
Implementation, history, perceptions, senses, and meanings	12		
	5	41.6	Sociohistorical evaluation of the creation and operation of the restaurants
	1	8.3	Theoretical saturation criterion
	1	8.3	Direct observation in all the cafeterias
	1	8.3	Phenomenological analysis
	3	25.0	Interview to explore the history, objectives, resources, and operations of the program
	3	25.0	Interview on the reasons for going to the restaurant
	1	8.3	Group counseling
	1	8.3	Focus group
Food handlers/workers	8		
	8	100.0	Sociodemographic Questionnaires: sex, age, self-classification of hair color, years of schooling, net family monthly income at minimum wages, marital status, residential situation, children, smoking habit, use of alcoholic beverages. Occupations: current position occupied, work time in the kitchen, use of lunch hours, body posture to perform tasks, courses taken in the food service area, training received in the workplace for the position, perception reported by the worker about the uncomfortable environmental conditions (temperature, noise, effort, light) and related to immediate cooking.
	5	62.5	Health Situation Questionnaire: general health status in comparison to people of your age, report of the presence of illnesses diagnosed by a doctor (arterial hypertension, diabetes mellitus, musculoskeletal illnesses related to work and gastritis), recent work accidents 12 months (cut, burn, electric shock, perforation by object and contusion)
	1	12.5	Classification of presence or absence of common mental disorders by means of responses to the abbreviated version of *General Health Questionnaire*
	2	25.0	Job Content Questionnaire (JCQ)
	3	37.5	Brazilian Food Insecurity Scale (EBIA)
	3	37.5	Nutritional Status
	1	12.5	Questionnaire of nutritional education in the restaurant
	1	12.5	Questionnaire of labor variables: position (store manager, chef, administrative assistant/nutritionist), production (kitchen assistant, cook, butcher), GSA/attendant (general services assistant and attendant); Working time in Community Restaurants
	1	12.5	24 h Recall (3 days)
	1	12.5	Self-assessment questionnaire for food handlers’ practices and knowledge of food safety
	1	12.5	Questionnaire to evaluate food safety knowledge of food handlers
Evaluation and monitoring	4		
	1	25.0	Normative evaluation
	1	25.0	Evaluation Matrix of Community Restaurants in Brazil
	1	25.0	Effectiveness
	1	25.0	Data Envelopment Analysis (DEA)
	1	25.0	Efficiency
Hygienic–sanitary quality	3		
	1	33.3	Application of the checklist based on the Manual of good food handling practices for restaurants and related Peruvian services
	1	33.3	Official Mexican Standard Application NOM-093-SSA1-1994 of Hygiene and sanitation practices in preparing food offered in Àjos establishments
	1	33.3	Semi-structured questionnaire on the type of service provided and contract, number of meals produced, type and composition of the menu, meal distribution system, opening hours of the restaurant, and composition of preparations.
	1	33.3	Sanitary inspection script (RIS) prepared based on Resolution RDC n. 216/2004 of ANVISA
	1	33.3	Support checklist for the implementation of Good Practices in Food Companies
Physical–functional planning	3		
	1	33.3	Calculation method by Teixeira et al. [83]
	1	33.3	List of equipment by sector of the restaurant
	1	33.3	Systematic observation using the method of Tobar and Yalour
	1	33.3	Application of a questionnaire on the characteristics of the Food Service (technical managers of the restaurants)
	1	33.3	Application of ANVISA’s sanitary hygienic aspects checklist (62 items)
Rest intake (Leftover/intake)	2		
	1	50.0	Evaluation using the Vaz method [84]
	1	50.0	Weighted Dish Waste Analysis

## Data Availability

The study did not report any data.

## References

[1] Adams K.J.U., Chirinos J.L. (2018). Prevalence of risk factors for metabolic syndrome and its components in community kitchen users in a district in Lima, Peru. Rev. Peru. De Med. Exp. Salud Publica.

[2] Andrade J.C.Y. (2016). Alimentación abundante, sana y barata’: Los restaurantes populares en Santiago (1936–1942). Cuad. De Hist..

[3] Balam-Gómez M., Uicab-Pool G., Uch-Puc P., Sabido-Barrera J. (2013). Evaluación de los comedores comunitarios en Tizimín, Yucatán, México: Percepciones y propuestas del personal y beneficiarios. Enfermería Univ..

[4] Dachner N., Gaetz S., Poland B., Tarasuk V. (2009). An ethnographic study of meal programs for homeless and under-Housed individuals in Toronto. J. Health Care Poor Underserved.

[5] Eppich S., Fernandez C.P. (2004). Study finds Chapel Hill, NC, soup kitchen serves nutritious meals. J. Am. Diet. Assoc..

[6] Furber S., Quine S., Jackson J., Laws R., Kirkwood D. (2010). The role of a community kitchen for clients in a socio-economically disadvantaged neighbourhood. Health Promot. J. Aust..

[7] Godoy K.C., Sávio K.E.O., Akutsu R.D.C., Gubert M.B., Botelho R.B.A. (2014). Perfile situação de insegurança alimentar dos usuários dos restaurantes populares no Brasil. Cad. De Saude Publica.

[8] Oyhenart E.E., Torres M.F., Quintero F.A., Luis M.A., Cesani M.F., Zucchi M., Orden A.B. (2007). Estado nutricional y composición corporal de niños pobres residentes en barrios periféricos de La Plata, Argentina. Rev. Panam. De Salud Publica/Panam. J. Public Health.

[9] Souza L.R., de Belik W. (2012). O planejamento da política de alimentação: Uma análise a partir dos casos do México, Brasil e Peru. Segurança Aliment. E Nutr. Camp..

[10] Contandriopoulos A.P., Champagne F., Denis J.L., Pineault R. (1997). Conceitos, Abordagens E Estratégias Para a Avaliação Em Saúde. HARTZ, ZMA., and SILVA, LMV. Orgs. Avaliação Em SaúDe: Dos Modelos Teóricos à Prática NA Avaliação de Programas E Sistemas de Saúde.

[11] Vieira-Da-Silva L.M., Furtado J.P. (2020). Health programs assessment: Continuities and changes. Cad. De Saude Publica.

[12] Pedrosa J.I.S. (2004). Perspectivas na avaliação em promoção da saúde: Uma abordagem institucional. Ciencia Saude Coletiva.

[13] Cohen E., Franco R. (2004). Avaliação de Projetos Sociais.

[14] de Hartz Z.M.A., de Silva L.M.V. (2005). Avaliação em Saúde: Dos Modelos Teóricos à Prática na Avaliação de Programas e Sistemas de Saúde.

[15] Cardoso M.D.O., Vieira-da-Silva L.M. (2012). Avaliação da cobertura da atenção básica à saúde em Salvador, Bahia, Brasil (2000 a 2007). Cad. De Saude Publica.

[16] Arksey H., O’Malley L. (2005). Scoping studies: Towards a methodological framework. Int. J. Soc. Res. Methodol. Theory Pract..

[17] Tricco A.C., Lillie E., Zarin W., O’Brien K.K., Colquhoun H., Levac D., Straus S.E. (2018). PRISMA extension for scoping reviews (PRISMA-ScR): Checklist and explanation. Ann. Intern. Med..

[18] Peters M.D.J., Godfrey C.M., McInerney P., Soares C.B., Khalil H., Parker D. (2015). The Joanna Briggs Institute Reviewers’ Manual 2015: Methodology for JBI Scoping Reviews.

[19] Aguiar O.B., Valente J.G., Fonseca M.D.J.M.D. (2010). Descrição sócio-demográfica, laboral e de saúde dos trabalhadores do setor de serviços de alimentação dos restaurantes populares do estado do estado do Rio de Janeiro. Rev. De Nutr..

[20] Amorim S.S., Silva M.M.S., Gomes S.T. (2007). Investimento Social e Perfil dos Usuários do Primeiro Restaurante Popular de Belo Horizonte-MG. Rev. Reun..

[21] Araújo F.Â.L.V., de Almeida M.I., Bastos V.C. (2007). Aspectos Alimentares e Nutricionais dos Usuários do ‘Restaurante Popular Mesa do Povo’. Alimentary and Nutritional Aspects of Users of the Popular Restaurant ‘Mesa do Povo’. Rev. Saúde E Soc..

[22] Assunção R.C.L.N., Bastos P.V., Silva B.P.L., Percegoni N., Mendes L.L., Binoti M.L. (2017). Perfil Socioeconômico, Demográfico E Alimentar Dos Usuários Do Restaurante Popular De Juiz De Fora–Mg. DEMETRA Aliment. Nutr. Saúde.

[23] Bento I.C., Hott Filgueiras J., Silva Abreu M.N., Cardoso Lisboa Pereira S., Gazzinelli M.F. (2016). Fatores associados às fases de comportamento alimentar de usuários dos restaurantes populares em Belo Horizonte/MG-Brasil. Rev. Port. De Saude Publica.

[24] Boas G.D.F.M.V., Botelho R.B.A., Akutsu R.D.C., Zandonadi R.P. (2021). Access to regional food in Brazilian community restaurants to strengthen the sustainability of local food systems. Int. J. Gastron. Food Sci..

[25] Botelho R.B.A., Akutsu R.D.C., Zandonadi R.P. (2019). Low-income population sugar (Sucrose) intake: A cross-sectional study among adults assisted by a brazilian food assistance program. Nutrients.

[26] Branquinho A.S., de Oliveira K.E.S., Akutsu R.D.C., da Silva E.F. (2015). Salud y perfil sócio-demográfi co de la clientela de restaurantes vinculados a programa social Brasileño. Rev. Chil. De Nutr..

[27] Canonico F.S., Pagamunici L.M., Ruiz S.P. (2014). Avaliação de sobras e resto-ingesta de restaurante popular do Município de Maringá-PR. Rev. UNINGÁ Rev..

[28] Caro F.B., Romero Hernández E.Y., Dennice González Fajardo K., Sánchez Viveros S., Torres R.M. (2018). Nivel de Seguridad Alimentaria en beneficiarios de Comedores Comunitarios del programa Cruzada Nacional contra el Hambre (México). Rev. Esp. Nutr. Comunitaria.

[29] Carrijo A.D.P., Botelho R.B.A., Akutsu R.D.C., Zandonadi R.P. (2018). Is what low-income Brazilians are eating in popular restaurants contributing to promote their health?. Nutrients.

[30] de Lima Costa B.V., Horta P.M., Jardim M.Z., do Carmo A.S., Ramos S.A. (2022). Obesity among government-backed economy restaurant workers in Belo Horizonte, Brazil. Rev. Bras. De Med. Do Trab..

[31] Costa B.V.D.L., Horta P.M., Ramos S.A. (2019). Food insecurity and overweight among government-backed economy restaurant workers. Rev. De Nutr..

[32] Darley C.L., Zanete M.E., Fochesatto A., Bonatto S. (2021). Perfil nutricional de usuários de um restaurante popular. Rev. Bras. De Obesidade Nutr. E Emagrecimento.

[33] Duarte I.A.E., Botelho R.B.A., Akutsu R.D.C. (2019). Regional Food Consumption in the Northeast of Brazil by the Low-Income Population. J. Culin. Sci. Technol..

[34] Falcão A.C.M.L., de Aguiar O.B., da Fonseca M.D.J.M. (2015). Association of socioeconomic, labor and health variables related to food insecurity in workers of the popular restaurants in the city of Rio de Janeiro. Rev. De Nutr..

[35] Fano T.J., Tyminski S.M., Flynn M.A.T. (2004). Evaluation of a Collective Kitchens Program Using the Population Health Promotion Model. Rev. Can. De La Prat. Et De La Rech. En Diététique.

[36] Fideles I.C., Akutsu R.D.C.C.D.A., Costa P.R., Costa-Souza J., Botelho R.B.A., Zandonadi R.P. (2020). Brazilian community restaurants’ low-income food handlers: Association between the nutritional status and the presence of non-communicable chronic diseases. Sustainability.

[37] Fideles I.C., Akutsu R.D.C.C.D.A., Barroso R.D.R.F., Costa-Souza J., Zandonadi R.P., Raposo A., Botelho R.B.A. (2021). Food insecurity among low-income food handlers: A nationwide study in Brazilian community restaurants. Int. J. Env. Res. Public Health.

[38] Fideles I.C., Akutsu R.D.C.C.D.A., Costa P.R.D.F., Souza J.C., Barroso R.D.R.F., Botelho R.B.A., Zandonadi R.P. (2022). Brazilian Food Handlers’ Years of Work in the Foodservice and Excess Weight: A Nationwide Cross-Sectional Study. Front. Public Health.

[39] Freedman M.R., Bartoli C. (2013). Food Intake Patterns and Plate Waste Among Community Meal Center Guests Show Room for Improvement. J. Hunger. Env. Nutr..

[40] Ginani V.C., Araújo W.M.C., Botelho R.B.A., Akutsu R.C.C.A., Zandonadi R.P. (2018). What is Offered by Public Foodservices for Low Income Population in Brazil is Adequate to Health Promotion Regarding Energy Density. J. Culin. Sci. Technol..

[41] Gobato R.C., Panigassi G., Villalba J.P. (2010). Identificação do perfil de usuários de um Restaurante Popular do Município de Campinas. Segurança Aliment. E Nutr..

[42] Godoy K., Sávio K.E.D.O., Akutsu R.D.C., Gubert M.B., Botelho R.B.A. (2017). Insegurança alimentar e estado nutricional entre indivíduos em situação de vulnerabilidade social no Brasil. Ciência E Saúde Coletiva.

[43] Gomes M.F.S., Pereira S.C.L., Abreu M.N.S. (2018). Factors associated with the self-rated health of elderly frequenters of low-budget community restaurants in Belo Horizonte. Cienc. E Saude Coletiva.

[44] Gonçalves M.P., Campos S.T.D., Sarti F.M. (2011). Políticas públicas de segurança alimentar no Brasil: Uma análise do Programa de Restaurantes Populares. Rev. Gestão Políticas Públicas.

[45] Hidalgo M.A.J., Chuquinaupa A.I.L., Luna J.M.F. (2009). Factores de riesgo de síndrome metabólico en mujeres socias de comedores populares del Cercado de Lima. Rev. Peru. De Cardiol..

[46] Kirchheim A.S., Garcia J.A., Baratto I. (2021). Implementação de um restaurante popular em um Município no interior do Paraná: Contribuições ao planejamento físico e funcional do local. Rev. Bras. De Obesidade Nutr. E Emagrecimento.

[47] Koh K.A., Bharel M., Henderson D.C. (2016). Nutrition for homeless populations: Shelters and soup kitchens as opportunities for intervention. Public Health Nutr..

[48] Lee J.H., Mccartan J., Palermo C., Bryce A. (2010). Process evaluation of Community Kitchens: Results from two Victorian local government areas Reaching Diverse Groups. Health Promot. J. Aust..

[49] Lima V.T., Gouveia P.M.T., Ribeiro A.A., Valdejane C., Souza S., Soares B.D. (2020). Perfil Antropométrico e de Doenças Crônicas não Transmissíveis de Idosos Frequentadores de Restaurantes Populares do Interior do Rio Grande do Norte-RN. Rev. Bras. De Ciências Da Saúde.

[50] Machado Í.E., Pereira S.C.L., Dias Júnior C.S., Abreu M.N.S., Borges A.M., Filgueiras J.H. (2014). Fatores associados ao excesso de peso em adultos usuários de restaurantes populares em Belo Horizonte, Brasil. Ciência E Saúde Coletiva.

[51] Mello A.G., Sales G.L.P., Jaeger L.M., Colares L.G.T. (2013). Estrutura físico-funcional de restaurantes populares do estado do Rio de Janeiro: Influência sobre as condições higiênico-sanitárias. Demetra Aliment. Nutr. Saúde.

[52] Minuzzi S.K., Alves M.K., Vicenzi K., Zanette C.D.A. (2018). Estado nutricional e perfil sociodemográfico de usuários de restaurantes populares em Caxias do Sul. Rev. Bras. De Obesidade Nutr. E Emagrecimento.

[53] Moraes S.D.R., Godoy K., Oliveira K.S.D. (2015). Diagnóstico da Insegurança Alimentar e do Estado Nutricional dos usuários dos restaurantes populares das Regiões Nordeste e Sul do Brasil. Tempus Actas De Saúde Coletiva.

[54] Mousa T.Y., Freeland-Graves J.H. (2019). Food security of food recipients of a food pantry and soup kitchen. Public Health Nutr..

[55] Oliveira J.T.C., Gabriel C.G., Machado M.L., Réos M.F., Soar C., Venske D.K.R. (2019). Government-Subsidized Restaurants as promoters of the realization of the Human Right to Adequate Food: Proposal of an evaluation model. Rev. De Nutr..

[56] Oliveira J.T.C.D., Gabriel C.G., Vasconcelos F.D.A.G.D., Machado M.L., Soar C., Fagundes A. (2020). Government-subsidized restaurants in Brazil: An evaluation within the framework of food and nutrition security. Rev. De Nutr..

[57] Poluha R.L., Motta C.C., Gatti R.R. (2016). Avaliação nutricional de refeições e análise de estrutura física em restaurante popular de Sorocaba-SP. Arch. Health Investig..

[58] Portella E.D.A., Basso C., Medina V.B. (2013). Perfil do usuário do restaurante popular da cidade de Santa Maria-RS. Discip. Sci. Saúde.

[59] Rauschenbach B.S., Frongillo E.A., Thompson F.E., Andersen E.J.Y., Spicer D.A. (1990). Dependency on soup kitchens in urban areas of New York State. Am. J. Public Health.

[60] Ribeiro A.A., Pessoa M.T.G., Maria S. (2017). Caracterização socioeconômica, estado nutricional e prevalência de insegurança alimentar em idosos usuários do restaurante popular de um município do nordeste brasileiro. Rev. Ciência Plur..

[61] Rosenblum A., Magura S., Kayman D.J., Fong C. (2005). Motivationally enhanced group counseling for substance users in a soup kitchen: A randomized clinical trial. Drug Alcohol Depend..

[62] Silva H.M.N.G.D., Pedelhes M.O., Costa A.D.J.B. (2016). Avaliação da efetividade de funções sociais de governo nas capitais estaduais. Bol. Governet De Adm. Pública E Gestão Munic..

[63] Sisson L.G., Lown D.A. (2011). Do soup kitchen meals contribute to suboptimal nutrient intake & obesity in the homeless population?. J. Hunger. Env. Nutr..

[64] Sobrinho F.M., Silva Y.C., Abreu M.N.S., Pereira S.C.L., Dias Júnior C.S. (2014). Fatores determinantes da insegurança alimentar e nutricional: Estudo realizado em Restaurantes Populares de Belo Horizonte, Minas Gerais, Brasil. Ciência E Saúde Coletiva.

[65] De Sousa J.R., Botelho R.B.A., Akutsu R.D.C.C.A., Zandonadi R.P. (2019). Nutritional Quality of Breakfast Consumed by the Low-Income Population in Brazil: A Nationwide Cross-Sectional Survey. Nutrients.

[66] Sousa J.R.D., Akutsu R.D.C., Zandonadi R.P., Botelho R.B.A. (2020). Breakfast characterization and consumption by low-income Brazilians: Food identity and regional food. Sustainability.

[67] Sousa M.S., Teixeira C.S.S., Souza J.C., Costa P.R.D.F., Zandonadi R.P., Botelho R.B.A., Akutsu R.D.C.C.D.A. (2021). Evaluation of the effectiveness of brazilian community restaurants for the dimension of low-income people access to food. Nutrients.

[68] Souza F.R.D., Dorr A.C., Saldanha P., Tonetto T.D.S., Guse J.C. (2014). Perfil dos usuários do restaurante popular da região centro do estado do Rio Grande do Sul. Rev. Eletrônica Em Gestão Educ. E Tecnol. Ambient..

[69] Souza C.V.S.D., Azevedo P.R.M.D., Seabra L.M.A.J. (2018). Food safety in Brazilian popular public restaurants: Food handlers’ knowledge and practices. J. Food Saf..

[70] Souza B.F.D.N.J.D., Marín-León L. (2013). Food insecurity among the elderly: Cross-sectional study with soup kitchen users. Food Insecurity Among The Elderly|679. Rev. Nutr..

[71] Zanete M.E., Darley C.L., Fochesatto A., Bonatto S. (2021). Hipertensão arterial sistêmica e fatores associados em usuários do restaurante popular de Caxias do Sul-RS. Rev. Bras. De Obesidade Nutr. E Emagrecimento.

[72] Araújo F.R.D., Araújo M.A.D.D., Batista M.P., Medeiros G.C.B.S., Souza F.J.V.D. (2016). Programa Restaurante Popular: Uma alternativa para promover o direito humano à alimentação adequada?. Emancipação.

[73] Braun M.B.S., Costa F.F.D. (2019). Impacto dos restaurantes populares na saúde e no desenvolvimento social dos usuários: O caso de Toledo (PR). Redes.

[74] Buttorff C., Trujillo A.J., Diez-Canseco F., Bernabe-Ortiz A., Miranda J.J. (2015). Evaluating consumer preferences for healthy eating from Community Kitchens in low-income urban areas: A discrete choice experiment of Comedores Populares in Peru. Soc. Sci. Med..

[75] Drinot P. (2005). Academy of American Franciscan History Food, Race and Working-Class Identity: Restaurantes Populares and Populism in 1930s Peru. The Americas.

[76] Hosseini H. (2017). Food insecurity and the use of soup kitchens among suburban elderly women in two counties in Pennsylvania. Humanomics.

[77] Kayman D.J., Gordon C., Rosenblum A., Magura S. (2005). “A port in a storm”: Client perceptions of substance abuse treatment outreach in a soup kitchen. J. Soc. Work Pract. Addict..

[78] Nunes F.R., Andrade B. (2013). O significado do Restaurante Popular de Maracanaú como Equipamento Público de Alimentação e Nutrição para as suas usuárias. Conhecer Debate Entre O Público E O Priv..

[79] Padrão S.M., Aguiar O.B.D. (2018). Restaurante popular: A política social em questão. Physis Rev. De Saúde Coletiva.

[80] Ramírez Y.P.G., Moreira R.R.D., Oliveira J.R.S.D. (2016). Avaliação de cardápio e identificação de alimentos funcionais: Estudo qualitativo de restaurante popular de Araraquara, São Paulo, Brasil. Segurança Aliment. E Nutr..

[81] Ramos S.A., Lima J.D.F.C., Carvalho A.C.M.D., Soares G.C., Batista J.A. (2020). Avaliação da qualidade das refeições servidas em um restaurante popular. HU Rev..

[82] Creed-Kanashiro H.M., Bartolini R.M., Fukumoto M.N., Uribe T.G., Robert R.C., Bentley M.E. (2003). Formative research to develop a nutrition education intervention to improve dietary iron intake among women and adolescent girls through community kitchens in Lima, Peru. J. Nutr..

[83] Teixeira S., Milet Z., Carvalho J., Biscontini T.M. (2007). Administração aplicada às unidades de Alimentação e Nutrição. Atheneu. Atheneu.

[84] Vaz C.S. (2006). Restaurantes: Controlando custos e aumentando lucros. Brasília Metha.

[85] Estrela C. (2018). Tipos de estudos. IN: ESTRELA, C. Metodologia científica. Ciência Ensino E Pesqui..

[86] Rouquayrol M.Z., Gurgel M. (2023). Rouquayrol–Epidemiologia e saúde. MedBook.

[87] Gerhardt T.E., Silveira D.T. (2009). Métodos de Pesquisa/ [organizado por] Tatiana Engel Gerhardt e Denise Tolfo Silveira; coordenado pela Universidade Aberta do Brasil–UAB/UFRGS e pelo Curso de Graduação Tecnológica–Planejamento e Gestão para o Desenvolvimento Rural da SEAD/UFRGS. Porto Alegre Ed. Da UFRGS.

[88] Bonita R., Beaglehole R., Kjellström T., Juraci A.C. (2010). Epidemiologia Básica.

[89] Lyles C.R., Drago-Ferguson S., Lopez A., Seligman H.K. (2013). Nutritional assessment of free meal programs in San Francisco. Prev. Chronic Dis..

[90] Carrijo A.D.P. (2013). Avaliação Do Consumo Alimentar Nos Restaurantes Populares Do Brasil. Master’s Thesis.

[91] George G.C., Milani T.J., Hanss-Nuss H., Kim M., Freeland-Graves J.H. (2004). Development and validation of a semi-quantitative food frequency questionnaire for young adult women in the southwestern United States. Nutr. Res..

[92] Hammond K., Mahan L.K., Stump S.E. (2002). Avaliação Dietética E Clínica. Krause–Alimentos, Nutrição E Dietoterapia.

[93] Institute of Medicine (2000). Dietary Reference Intakes. Applications in Dietary Assessment: A Report of the Subcommittees on Interpretation and Uses of Dietary Reference Intakes and the Standing Committee on the Scientific Evaluation of Dietary Reference Intakes, Food and Nutrition Board, Institute of Medicine.

[94] Savio K.E.O., Costa T.H.M.D., Miazaki É., Schmitz B.D.A.S. (2005). Avaliação do almoço servido a participantes do programa de alimentação do trabalhador. Rev. Saude Publica.

[95] Brasil M.D., SaÚde S.D.V.E.S., Departamento de Vigilância de Doenças e Agravos não Transmissíveis e Promoção de Saúde (2015). Vigitel Brasil 2014: Vigilância de Fatores de Risco e Proteção para Doenças Crônicas por Inquérito Telefônico.

[96] CDC (2012). National Center for Chronic Disease Prevention and Health Promotion Division of Nutrition and Physical Activity. Can eating fruits and vegetables help people to manage their weight? Research to Practice Series, no. 1 CDC. http://www.cdc.gov/nccdphp/dnpa/nutrition/pdf/rtp_practitioner_10_07.pdf.

[97] Brasil. Programa de Alimentação do Trabalhador (2006). Parâmetros nutricionais do PAT. Portaria Interministerial no. 66, de 25 de agosto de 2006 (Parâmetros Nutricionais do PAT). https://www.normaslegais.com.br/legislacao/portariainterministerial66.htm.

[98] WHO. World Health Organization (2003). Diet, Nutrition and The Prevention of Chronic Diseases.

[99] WHO. World Health Organization (2018). Diet, Nutrition and The Prevention of Chronic Diseases.

[100] Camargo E.B., Botelho R.A. (2005). Técnica Dietética: Seleção E Preparo de Alimentos–Manual de Laboratório.

[101] FDA. Federal Drug Administration (2016). Diet and lifestyle recommendations revision 2006: A scientific statement from the American Heart Association nutrition committee. Circulation.

[102] FAO. Food and Agriculture Organization (2017). El Estado de la Seguridad Alimentaria Y la Nutrición en El Mundo 2017. Fomentando la Resiliencia en Aras de la Paz Y la Seguridad Alimentaria.

[103] Segall-Corrêa A.M., Marin-Leon L. (2009). A Segurança Alimentar no Brasil: Proposição e Usos da Escala Brasileira de Medida da Insegurança Alimentar (EBIA) de 2003 a 2009. Campinas.

[104] Bickel G., Nord M., Price C., Hamilton W., Cook J. Measuring Food Security in the United States Guide to Measuring Household Food Security Revised 2000. http://www.fns.usda.gov/oane.

[105] FAO. Food and Agriculture Organization (1990). Manual de Uso Y Aplicación de Escala Latinoamericana Y Caribeña de Seguridad Alimentaria.

[106] Yin R.K. (2005). Introducing The World Of Education: A Case Study Reader.

[107] Brasil I brasileiro de opinião público (IBOPE) (2005). Pesquisa de Opinião Pública Com Usuários de Restaurantes Populares. www.mds.gov.br.

[108] Federation International Diabetes (2006). The IDF Consensus Worldwide Definition of The Metabolic Syndrome.

[109] Malta D.C., Bernal R.T.I., Andrade S.S.C.D.A., da Silva M.M.A., Velasquez-Melendez G. (2017). Prevalence of and factors associated with self-reported high blood pressure in Brazilian adults. Rev. Saude Publica.

[110] WHO. World Health Organization (2002). Food and Agricultural Organization of the United Nations. Diet, Nutrition and the Prevention of Chronic Disease.

[111] WHO. World Health Organization (1995). Physical. The Use and Interpretation of Anthropometry.

[112] Dobson K. (1988). Handbook of Cognitive–Behavioral Therapies.

[113] Silva A.P., Barros C.R., Nogueira M.L.M., Barros V.A.D. (2007). ‘Conte-me sua história’: Reflexões sobre o método de História de Vida ‘Tell me your history’: Reflections about the Life History method. www.fafich.ufmg.br/mosaico.

[114] Spindola T., Santos R.D.S. (2003). Trabalhando com a história de vida: Percalços de uma pesquisa(dora?). Rev. Da Esc. De Enferm. Da USP.

[115] Demo P. (1985). Introdução a Metodologia.

[116] Minayo M.C.D.S. (1996). O Desafio Do Conhecimento: Pesquisa Qualitativa Em Saúde. HUCITEC.

[117] Souza L.K.D. (2020). Recomendações para a Realização de Grupos Focais na Pesquisa Qualitativa. PSI UNISC.

[118] Sordini M.V. (2023). Prácticas de reciprocidad en comedores comunitarios: Entre el amor, la confianza y la esperanza. Trab. Soc..

[119] Brasil. Rede Integrada de Segurança Alimentar E Nutricional (2011). Equipamentos Públicos de Segurança Alimentar E Nutricional.

[120] Gomes A.D.O., Lopes L.P.F., Zancan C., Neto M.C.D.L. (2017). Variáveis correlacionadas com a produtividade de juízes da primeira instância da Justiça Estadual de Minas Gerais. Sist. Gestão.

[121] Karasek R., Brisson Q., Kawakami N., Houtman I., Bongers P., Amick B. (1998). The Job Content Questionnaire (JCQ): An Instrument for Internationally Comparative Assessments of Psychosocial Job Characteristics. J. Occup. Health Psychol..

[122] Andersen L.L., Izquierdo M., Sundstrup E. (2017). Overweight and obesity are progressively associated with lower work ability in the general working population: Cross-sectional study among 10,000 adults. Int. Arch. Occup. Env. Health.

[123] ANVISA. Agência Nacional de Vigilância Sanitária (2004). Resolução RDC no. 216. Dispõe sobre Regulamento Técnico de Boas Práticas para Serviços de Alimentação. Agência Nacional de Vigilância Sanitária Diário Oficial da União.

[124] WHO. World Health Organization (2013). Physical Status: The Use and Interpretation of Anthropometry.

[125] Santos M.J., Nogueira J.R., Patarata L., Mayan O. (2008). Knowledge levels of food handlers in Portuguese school canteens and their self-reported behaviour towards food safety. Int. J. Env. Health Res..

[126] Wilbert M.D., D’Abreu E.C.C.F. (2013). Eficiência dos gastos públicos na educação: Análise dos municípios do estado de alagoas. Adv. Sci. Appl. Account..

[127] Patrus A. (2007). A Política Social e a Resposta à Globalização. https://repositorio.enap.gov.br/jspui/bitstream/1/4162/1/Livro_Teorias%20e%20An%C3%A1lises%20sobre%20Implementa%C3%A7%C3%A3o%20de%20Pol%C3%ADticas%20P%C3%BAblicas%20no%20Brasil.pdf.

[128] Sano H., Filho M.J.F.M. (2013). As Técnicas de Avaliação Da Eficiência, Eficácia E Efetividade NA Gestão Pública E Sua Relevância Para O Desenvolvimento Social E Das ações Públicas. Desenvolv. Em Questão.

[129] Perú (2008). Manual de Buenas Pricticas de Manipulación de Alimentos Para Restaurantes Y Servicios Afines Peruano.

[130] México (1995). Norma Ofcial Mexicana NOM-093-SSA1-1994 de Pricticas de hiJiene y sanidad en la preparación de alimentos que se ofrecen en establecimientos Àjos. Dirección General de Control Sanitario de Bienes y Servicios.

[131] ANVISA. Agência Nacional de Vigilância Sanitária (2002). Resolução–RDC no. 275, de 21 de Outubro de 2002. Estabelece Procedimentos Operacionais Padronizados que Contribuam para a Garantia das Condições Higiênico-Sanitárias Necessárias ao Processamento/Industrialização de Alimentos, Complementando as Boas Práticas de Fabricação. Diário Oficial da União Brasília.

[132] Saccol A.L.D.F. (2007). Sistematização de Ferramentas de Apoio Para Boas Práticas em Serviços de Alimentação. Master’s Thesis.

[133] Brasil (2007). Manual de Implantação de Restaurante Popular.

[134] Maynard D.D.C., Zandonadi R.P., Nakano E.Y., Raposo A., Botelho R.B.A. (2021). Green restaurants assessment (Grass): A tool for evaluation and classification of restaurants considering sustainability indicators. Sustainability.

[135] Santiago L.A., Ramos S.A., Batista J.A. (2022). Análise da produção de resíduos e de ações de sustentabilidade em um Restaurante Popular do município de Belo Horizonte. Res. Soc. Dev..

